# COVID-19 and digital disruption in UK universities: afflictions and affordances of emergency online migration

**DOI:** 10.1007/s10734-020-00561-y

**Published:** 2020-06-04

**Authors:** Richard Watermeyer, Tom Crick, Cathryn Knight, Janet Goodall

**Affiliations:** 1 School of Education, University of Bristol, Helen Wodehouse Building, 35 Berkeley Square, Bristol, BS8 1JA UK; 2 School of Education, Swansea University, Singleton Park, Swansea, SA2 8PP UK

**Keywords:** COVID-19, UK higher education, Online learning, teaching and assessment, Digitalisation of universities, Academic profession

## Abstract

COVID-19 has caused the closure of university campuses around the world and migration of all learning, teaching, and assessment into online domains. The impacts of this on the academic community as frontline providers of higher education are profound. In this article, we report the findings from a survey of *n* = 1148 academics working in universities in the United Kingdom (UK) and representing all the major disciplines and career hierarchy. Respondents report an abundance of what we call ‘afflictions’ exacted upon their role as educators and in far fewer yet no less visible ways ‘affordances’ derived from their rapid transition to online provision and early ‘entry-level’ use of digital pedagogies. Overall, they suggest that online migration is engendering significant dysfunctionality and disturbance to their pedagogical roles and their personal lives. They also signpost online migration as a major challenge for student recruitment, market sustainability, an academic labour-market, and local economies.

## Introduction

The societal impact of COVID-19 is almost incalculable. It has affected, and continues to affect, profound social suffering and deep economic hardship. While indiscriminate in terms of whom it infects, it has punished most gravely society’s most vulnerable and less fortunate (The Lancet 2020; von Braun et al. 2020). Worse now, it appears that the virus must be tolerated on an indefinite basis (Kissler et al. 2020).

For those working in an educational context, the immediate impact of COVID-19 has been lockdown and the enforced closure of schools, colleges and universities. Yet, this has not meant the cessation of all learning, teaching and assessment (LTA)—though we acknowledge that many forms of assessment have been suspended. Instead, LTA has in many instances transitioned with great haste and speed into online domains. In the more specific context of universities, academics as higher education providers have been thrust headlong into providing for their students exclusively via a digital interface (cf. Kernohan 2020). For many, this has been an unusual, disorienting and even an unwelcome experience, as the following discussion illuminates. However, perhaps more than this, the experience of rapid online migration of LTA has revealed much of the deficiencies of the higher education sector and much perhaps of what needs to change in universities. Certainly, this is relevant to scrutinising their educational provision and pedagogical investment too frequently confounded by the performative subsumptions of higher education as a prestige economy (Blackmore 2015; Blackmore and Kandiko 2011), a veneration of rankings (Peters 2019), and complicity with crude and inexact indicators of what counts (cf. Collini 2017; Watermeyer 2019). COVID-19 has thus not only forced change but revealed quite how much such change is overdue. Concurrently, much as the COVID-19 crisis, like nothing else before it, is articulating the severity of social and economic inequality and fomenting also a reconsideration, even refutation of the kinds of social stratification and democratic infringements (cf. Zuboff 2019) committed by global capitalism—and equally mobilising the restitution and reclamation of the public sphere—so too is it magnifying the egregious faults and failures of universities (as explicitly, even now unapologetically neoliberalised organisations) and with such force that they may no longer be hidden or defended.

For what seems an age and too long now, higher education commentators have talked of the future of the university and the university reimagined—especially as freed from the binds of neoliberal excess (cf. Izak et al. 2017). Endless pages have been devoted to the critique of why higher education either does not work, does not work as it should, or may no longer need to work in the context especially of rapid technological innovation and labour market transformation (cf. Aoun 2017; Arvanitakis and Hornsby 2016; Marshall 2018). Yet, now in the face of COVID-19 and emergency remote LTA, the immediate and perhaps long-term future of higher education appears inextricably linked to what others in recent years have discussed as its digital transformation (cf. Castañeda and Selwyn 2018; Henderson et al. 2017; Macgilchrist et al. 2020; Marshall 2018; Selwyn 2016, 2017; Tømte et al. 2019; Williamson 2020).

A need thus to take the pulse of universities or more specifically of academics as the rank and file of teaching (and research) in such uncertain, challenging and changing times is exigent—if only in helping to guide the impact of such digital transformation—no matter how ephemeral it may turn out to be; there are many agnostics—in constructive and crucially, humane ways. There are clear sensitivities and alleged ethical traps in doing so. Yet, mapping this terrain is vital. We cannot just wait to see what happens (or does not). To do so would be a neglect of responsibility in not only guiding but also claiming agency over the next steps of change, where such steps already are profoundly affecting not only the role and identity of academics as higher educators but also their welfare and livelihood. Moreover, to do nothing would be falling prey to the languidness that has dogged many universities and caused their arrest in rethinking their role and relevance—and how they support their communities—in what is now incontrovertibly a digital age and what others have described as the era of the fourth industrial revolution (Schwab 2017).

For such reasons, we undertook a consultation of UK academics and their perspectives as individuals in the very mix of online transitioning in the wake of COVID-19, and what they are identifying and forecasting respectively as its immediate and prospective impacts. The discussion that follows is based upon the perspectives of *n* = 1148 academics drawn from across a career hierarchy, the major disciplines, and different kinds of institutions, and what they recognise to be the major consequences of COVID-19 and a transition to online LTA. Their accounts document the hopes and fears of the higher education community in the face of seismic and, as may prove to be, inalterable shifts. While the vast majority of respondents tend towards a negative view of online migration, which are represented in the following discussion as ‘afflictions’, there were some, albeit a minority, who spoke of its ‘affordances’ and who adopted a far more positive and optimistic tone in deliberating the impact of COVID-19 on higher education.

## Methodology

### Survey design

The survey was designed to gather information on how the education workforce was responding to the move to online learning teaching and assessment. The survey was constructed and disseminated using the online tool Qualtrics. When the participants commenced the survey, they were given information about the purpose of the research and were informed of their anonymity and right to withdraw. The following questions then gathered information about relevant demographic information (country, discipline, gender, years of experience, position). These were followed by closed-ended questions designed to identify respondents’ feelings and experiences with online LTA. Finally, respondents were asked to ‘provide any comments of how the online learning and teaching changes brought in as a response to COVID-19 will impact upon’ followed by ‘your role’, ‘your institution’ and ‘your sector of education’.

### Survey dissemination

The survey was initially piloted on a subsample of the population. Pilot respondents were asked to comment upon the clarity of the survey questions and the functionality of the survey as a whole. In light of these comments, changes were made before the survey was disseminated to the wider education workforce. The target population for this survey was those who worked in an education setting. Those who did not meet this criterion were excluded from analysis post hoc. This paper focuses on respondents who said that they worked in a higher education (university) setting.

The survey was distributed through our own professional networks and utilising online platforms such as social media (Twitter and LinkedIN, especially) and emails via relevant distribution lists, for instance the UK’s University and College Union and British Educational Research Association, Higher Education Special Interest Group. Thus, respondents were recruited through a convenience sample using snowballing from the initial targeted contacts.

While not designed to be representative, the survey aimed to gain insight into key trends in the perspectives of the education workforce. After removing those who did not meet the target population, the survey gathered *n =* 1148 responses from academics—a majority who were female—working across the four home nations of the UK, with academics from England having by far the largest sector of the four, being most represented. Table 1 shows the HE respondent demographics. Due to the distribution method, we cannot calculate the response rate. However, of those who started the survey, 84.9% completed it.

**Table 1:** Respondent demographics

Variable	Category	*n*	%
Country	England	907	79.1
	Wales	110	9.6
	Scotland	104	9.1
	Northern Ireland	25	2.2
Gender	Male	457	39.7
	Female	655	57.1
	Prefer to self-describe	11	1.0
	Prefer not to say	24	2.1
Disciplinary area	Medicine and dentistry	10	1.0
	Subjects allied to medicine	53	5.4
	Biological sciences	39	4.0
	Veterinary science	3	0.3
	Agriculture and related subjects	1	0.1
	Physical sciences	40	4.1
	Mathematical sciences	29	3.0
	Computer science	119	12.2
	Engineering and technology	46	4.7
	Architecture, building and planning	12	1.2
	Social science	155	15.9
	Law	33	3.4
	Business and administrative studies	99	10.2
	Mass communications and documentation	14	1.4
	Languages	55	5.7
	Historical and philosophical studies	65	6.7
	Creative arts and design	67	6.9
	Education	133	13.7
Position	Lecturer (Assistant Professor)	265	25.5
	Senior Lecturer/Reader (Associate Professor)	470	40.9
	Professor	159	15.3
	Graduate Teaching Assistant/Fellow	28	2.7
	Teaching Fellow	62	6.0
	Academic related	54	4.7
Years working in sector	0–5	202	18.1
	6–10	185	16.5
	11–15	217	19.4
	16–20	157	14.0
	21–25	164	14.7
	26+	194	17.3
Employment status	Part-time	233	21.0
	Full-time	876	79.0
Contract	Fixed term	131	11.7
	Permanent	932	83.4
	Zero-hours	33	3.0
	Other	19	1.7
Total		1148	100

The survey was launched on the 26 March and remained open for 4 weeks.

### Data analysis

Quantitative data were derived mainly through Likert and slider-scale questions. Prior to analysis, for ease of interpretation, five-point Likert scales (strongly agree, agree, neither agree nor disagree, disagree, strongly disagree) were recoded into binary ‘agree’ and ‘disagree’ variables. Data were analysed using univariate and chi-square tests in order to understand overall participant views, along with establishing whether there were significant differences between demographic groups.

The open-ended questions were analysed using grounded theory (Strauss and Corbin 1994; Bryant and Charmaz 2010); responses were read several times, coding applied to each individual group of responses then the entire dataset was re-read and coding adjusted as required.

## Headline findings

### Preparedness

**Fig. 1:**
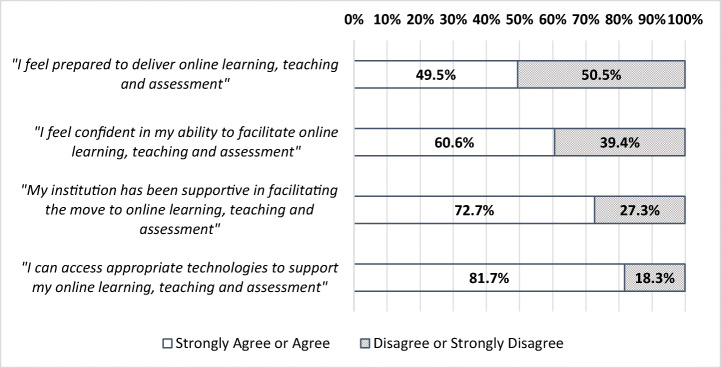
Agreement to statements on LTA

Respondents were asked how much they agreed with the statement ‘I feel prepared to deliver online learning, teaching and assessment’. Out of all of the HE respondents, 49.5% either strongly agreed or agreed with this statement (Fig. 1).

Table 2 shows those in computer sciences (66%) and education (64.2%) were more likely to agree whilst those in biological sciences (27.8%) and languages (30.4%) were less likely to agree that they felt prepared: *X*^2^(16) = 40.96, *p* = 0.001. Those in academic related roles (e.g. academic management, librarian, etc.) (73.2%) were also significantly more likely to agree that they felt prepared: *X*^2^(5) = 12.8, *p* = 0.03.

**Table 2:** Sector perceptions by discipline and career position

		Preparedness	Confidence	Support	Access
		*N* (%)*	*N* (%)*	*N* (%)*	*N* (%)*
Discipline	Subjects allied to medicine	23 (51.1%)	24 (57.1%)	23 (63.9%)	34 (77.3%)
	Biological sciences	*10 (27.8%)*	15 (55.6%)	23 (74.2%)	26 (81.3%)
	Physical sciences	19 (59.4%)	21 (65.6%)	23 (74.2%)	26 (81.3%)
	Mathematical sciences	11 (45.8%)	13 (59.1%)	15 (65.2%)	17 (77.3%)
	Computer science	**66 (66.0%)**	**72 (75.8%)**	**82 (86.3%)**	95 (94.1%)
	Engineering and technology	20 (67.1%)	22 (68.8%)	25 (80.6%)	30 (83.3%)
	Social science	52 (42.6%)	*57 (51.4%)*	75 (67.0%)	98 (80.3%)
	Law	16 (53.3%)	17 (60.7%)	19 (73.1%)	22 (84.6%)
	Business and administrative studies	35 (44.9%)	44 (58.7%)	49 (77.8%)	55 (76.4%)
	Languages	*14 (30.4%)*	19 (48.7%)	*22 (53.7%)*	31 (73.8%)
	Historical and philosophical studies	22 (42.3%)	25 (51.0%)	35 (68.6%)	41 (75.9%)
	Creative arts and design	26 (50.0%)	30 (66.7%)	*25 (52.1%)*	41 (74.5%)
	Education	**68 (64.2%)**	**74 (72.5%)**	**87 (85.3%)**	90 (84.9%)
Career Position	Lecturer (Assistant Professor)	96 (44.4%)	116 (58.6%)	138 (72.3%)	173 (80.1%)
	Senior Lecturer/Reader (Associate Professor)	203 (50.9%)	228 (62.0%)	243 (69%)	305 (79.6%)
	Professor	60 (46.2%)	73 (58.9%)	**102 (79.7%)**	101 (83.5%)
	Graduate Teaching Assistant/Fellow	–	–	13 (72.2%)	19 (95.0%)
	Teaching Fellow	23 (46.9%)	26 (56.5%)	*29 (55.8%)*	44 (86.3%)
	Academic related	**30 (73.2%)**	28 (71.8%)	**37 (92.5%)**	39 (88.6%)
Total		451 (49.5%)	516 (44.9%)	606 (72.7%)	727 (81.7%)

Those in bold had a z score of + 1.96 meaning that this category were significantly more likely than expected to agree with the statement, those in italics has a z score of − 1.96 meaning that this category was significantly less likely than expected to agree with the statement. Cells with bellow 10 cases are not reported

*Number and percent of those who agreed with the statement, compared to those who disagreed with the statement

### Confidence

In addition to feeling prepared, respondents were asked how much they agree with the statement ‘I feel confident in my ability to facilitate online learning, teaching and assessment’. Of respondents, 60.6% either strongly agreed or agreed with this statement (Fig. 1).

Table 2 shows that those from computer sciences (75.8%) and education (72.5%) were more likely to agree while those from social sciences (51.4%%) were more likely to disagree: *X*^2^(16) = 26.6, *p* = 0.05).

### Support from institution

In response to the statement ‘My institution has been supportive in facilitating the move to online learning, teaching and assessment’, 72.7% of respondents agreed or strongly agreed (Fig. 1).

Those in computer sciences (86.3%) and education (85.3%) were more likely to agree while languages (53.7%) and creative arts and design (52.1%) were less to agree: *X*^2^(17) = 45.9, *p* < 0.001 (Table 2). Responses to this statement also differed by career position whereby professors (79.7.1%) and those in academic related positions (92.5%) were significantly more likely to agree with the statement, whilst teaching fellows (55.8%) were significantly less likely to agree: *X*^2^(5) = 20.4, *p* = 0.001.

### Access

Respondents were asked the extent to which they agreed to the statement ‘I can access appropriate technologies to support my online learning, teaching and assessment’. Of respondents, 81.7% either strongly agreed or agreed with this statement.

### Impact on workload

**Fig. 2:**
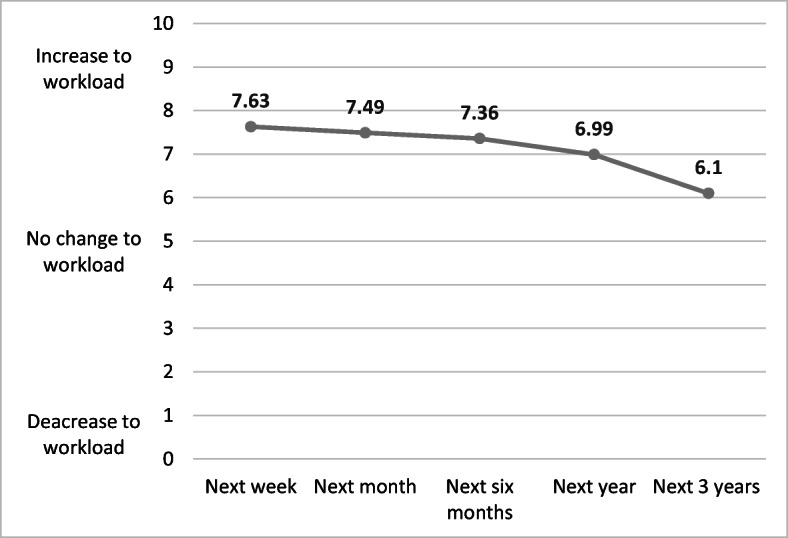
Perceived impact on workload

Respondents were asked on a scale of 0 (decrease to workload) to 10 (increase to workload) ‘How much do you think the recent changes, as a response to COVID-19, will impact on your workload in the next’ followed by ‘week’, ‘month’, ‘six months’, ‘year’ and ‘three years’. Figure 2 presents the average responses which show that the sector believe that the changes will increase workload as whole over the next 3 years—with the largest increase to workload expected in the next week and month.

### Afflictions

As already reported, our data reveals significant variation in terms of how academics in the UK perceive preparedness and confidence for total online migration of all LTA, with major implications for the competitiveness and thus also sustainability of universities within a global student marketplace. Respondents as a whole recognised that institutions already invested in digital education and with a preexisting and developed infrastructure and capacity to deliver LTA online would be advantageously positioned to attract greater numbers of students. Our respondents repeatedly talked of the *Open University*^1^ as being in such terms an exemplar and market-leader and also an institution for which total online migration of LTA would require, unlike many other institutions, little adjustment.

It was felt across the board that the future of student recruitment—and importantly not only in the immediate context of 2020/2021 admissions—rested with the extent of universities’ digital offering. While many respondents expressed their concerns that a turn to online LTA would damage the attractiveness (and holism) of a university education, others reflected that it might become more economically appealing, where online provision would do away with the need for expensive physical relocation—a cost experienced most heavily by international student cohorts: ‘It will be massively appealing to those abroad who can afford tuition but not living costs’. Correspondingly, many of our respondents questioned the sustainability of high tuition fees charged by many universities and claimed that going online would enable overall cost reductions or rather would reduce what students would be prepared to pay for, especially given how much learner content is already freely available: ‘Some things are now available for free, that are expensive. This will have an impact on peoples’ position, and their attitude of paying for teaching’. It was generally felt therefore that institutions would no longer be able to simply rely on the quality of their branding for student capture but have to explicitly demonstrate the quality of their digital offering to remain competitive—and even more so given the challenge presented by more technologically adept private higher education providers:

*We will see that some students - especially international students - will be less willing to pay a premium (whether directly in tuition or indirectly in accommodation costs etc) for the prestige and reputation of an institution and be more aware of the importance of its information technology infrastructure and the quality of its virtual learning environment.*

The impact of digitalisation on the marketisation of higher education would it was felt be especially profound in the content of opening up a ‘weapons race to see who can sustain the distance learning side of course provision’. For those playing digital catch-up, the threat of losing out on student numbers and thus remaining economically viable, was considered to be most acute. Beyond those institutions already technologically committed, arguably only the most prestigious, established and wealthiest institutions might survive such market change. Especially so given the wider financial implications of universities going online, with much of their additional offering in the terms of accommodation, leisure and recreation and other forms of social provision becoming obsolete. Many of the numerous staff involved in the delivery of these services would, therefore, face redundancy as too it was feared would many academic staff, no longer necessary to servicing the in-person demands of a massified student body. The university experience would thus also fundamentally alter for many students, especially undergraduates, who would no longer experience a unique life-stage of social learning and development. The digitalisation of higher education would also bring about the emptying of university campuses, the abandoning (and necessary repurposing) of halls of residence, student unions and the like. So too would local economies benefitting from student patronage massively suffer. A recent trend in the UK of investment in private student accommodation would seem one obvious casualty. At the same time, recent emphasis on universities’ internationalisation and student exchange especially would weaken as too the contribution to countries’ GDP made by international students. By way of example, the net economic contribution of the UK’s 2015/2016 cohort of international students, has been calculated to be $20.3 billion (Higher Education Policy Institute 2018)—no trifling amount.

This kind of hollowing out, it was felt by some, represented a further blow to an ideal of a university education and its further deterioration in a marketised context:

*Higher ed is already trying to turn its product into an inferior mass produced product. How can this do anything but help that along?*

Others still considered a digital turn to be corrosive to core academic values and the very mission of the university:

*The communitarian values of the academy may be diminished in the long term and the uniqueness of our shared enterprise of seeking truth along with them.*

Some of our respondents, considered that where online migration was ‘reducing the amount of perceived value-added activity the university provides’, that this would result in ‘increasing the demands of students-as-consumers’ and therefore further intensify performance-evaluation regimes—and unassailability of a doctrine of ‘competitive accountability’ (Watermeyer 2019)—in universities and correspondingly diminish academics’ creative and critical freedoms.

*The central university will exert greater control over academics to ensure uniformity and regulation of changes to teaching and assessment. I will be less free to recommend and to employ innovative pedagogies.*

In fact, some of our respondents even spoke of how students’ disinclination towards online LTA would translate almost putatively into an aspect of intensifying performance evaluation:

*Our students will now expect flexible or online delivery, even though they loathe it, in the name of convenience. They will use teaching evaluation surveys to punish staff for the fact that they loathe it.*

Many respondents spoke of enforced online migration in the context of pedagogical dumbing-down and of their deprofessionalisation—some also making a link to the kinds of deskilling and proletarianisation in higher education caused by the ubiquity of fiscal rationalisation and market determinism: ‘in making education mechanical and impersonal, it’ll help bring about the neoliberals’ nirvana, the end of any real expertise and personal interaction’.

For the overwhelming majority of our respondents, a move to online LTA was viewed negatively and based on a view that their pedagogical praxis had been reduced to the fulfilment of rudimentary technical functions, functions moreover that might easily be automated. Furthermore, it was felt that whatever pedagogical role might be claimed by academics was now purely didactic, transmissional and therefore regressive. Certainly, it would appear contrary to current policy directions or be that market trends and a professionalisation agenda for academics as teachers—with an investment in soft-credential building seemingly justifying high tuition costs and buttressing claims of teaching excellence. In terms more honest and relevant to a teaching philosophy, online migration would, however, appear in these early stages to signal the abandonment of higher education as a socially immersive and participatory learning experience and its return to the lectern. Yet, these views should be understood as nascent experiences of digital education shaped under the panic and duress of emergency conditions:

*I feel like I may as well be a technician rather than an instructor. I’m just posting stuff online and trying to keep up with far too many online discussions.*

*I am becoming a more disembodied and depersonalised purveyor of education. My role will necessarily be more to transmit academic information rather than teach and model an academic ethos.*

These views, however immediate in their response to an emergency-footing and arguably less than well-informed of the potential of digital education, are no less damaging where academics as frontline providers of higher education, appear on the evidence of these accounts at least, antagonistic to it. They also very much reflect a general fear habitually exhibited by those who perceive technology as not so much advantageous as debilitative to their occupational welfare (cf. Aoun 2017) and furthermore, online education as no more than a further iteration of neoliberal reform (Feenberg 2017). Moreover, they signpost how academics’ pedagogical praxis in the context of rapid online migration is not only being destabilised and transformed by technical needs but also significant extension of the pastoral role.

A great number of our respondents spoke of how the pastoral side of their job had rapidly and substantially increased, and that they were now committing significant time in a counselling capacity. Some framed their pastoral role in the context of acting as student support substitutes:

*It has become much more pastoral; helping students who are upset and frightened has become a much bigger part of my role, as they turn to tutors for help. Previously student support services would have filled this need.*

At this time, we are unaware of—and our survey did not capture—the extent to which standard student support services remain open and available to students. However, these accounts suggest that academic tutors are operating as first and perhaps only point of contact and are thus assuming levels of responsibility for their students’ welfare, that exceed their expertise and training, what might be reasonably asked of them, and moreover their contractual and thus legal obligations. Respondents discussed the extent to which they were now dealing explicitly with students’ mental health problems and concerns far exceeding the jurisdiction of their studies:

*I am having to deal with an enormous increase in students contacting me with mental health problems resulting from a lack of clarity over their learning, and wider anxieties about the current situation.*

Undertaking this duty, for the vast majority without or with at best limited mental health training, was also felt to be problematic at a time where academics were having to adjust to the challenges of working from home whilst accommodating general workload intensification. They also pointed to technological deficiencies—and as we have also reported of feeling ill-prepared, lacking confidence and institutional support—and intimated the vocational nature of academics and academics as ‘perfectionists’:

*The increase to workload - at a time when colleagues’ home lives do not facilitate long working hours - is incredibly difficult. Add in (inevitable) technology glitches, a stressed student body and the inherently perfectionist nature of academics and you have a tinder box of stress.*

Some also directly spoke of work intensification due to online rerouting of LTA and of reduced opportunities for one-on-one discussions in online contexts, resulting in additional and extended student consultations:

*I'm doing far more one-to-one consultations with students (the kind that would normally sometimes happen in office hours, but more often in a quick chat at the start or end of a seminar) than has normally been part of my role - I think because the forced group element of an online seminar doesn't leave any room for private conversations.*

Attending to the needs of huge groups of students in an online and yet home-based environment was felt by respondents to be especially challenging. In fact, working from home was viewed as actually contributing to work intensification and the erosion of work-life balance. Respondents alluded to the collapsing of customary parameters separating work from personal lives and the timelessness of being online in a home setting exacerbating compulsive working and ‘hyper-professionality’ as traits common to academics (Gornall and Salisbury 2012):

*The change to working from home has involved amassing a huge amount of information to teaching online as opposed to face to face teaching. This is especially pertinent to my role as a module leader for a module with 400 students currently. Rather than being able to address groups of students, I am now having to post notices on the internet or respond to individual queries which takes more time. I think there is also a temptation to carry on working for longer hours than normal as my 'office' is in my sitting room and it is too easy to ‘just send one more email’.*

The challenge of working from home and servicing the escalated and seemingly endless pastoral needs of students—many located across multiple international time-zones yet no less seeking continuous access to their tutors—was seen to be further compounded in the case of academics with home care responsibilities and child dependents, especially. Respondents spoke of their frustration and even their resentment at being unable to adequately cater for both and intimated the invasiveness of, and exhaustion suffered from an expectation (primarily from their institutions) of being digitally, and therefore around-the-clock accessible to students and the impact thereof in terms both of their personal wellbeing and professional development. The overexposure in such terms of academics with parental responsibilities will surely, we conjecture, have a professional impact, not least especially on research productivity. We might well anticipate a dip in global publishing as a consequence with many scientific journals—perhaps more so than universities—exercising social responsibility in recognising the unprecedented LTA demands being faced by academics and easing requests made of academics as peer-reviewers.

Yet, despite what may be quite welcome signs of the deacceleration of academic publishing, it may be female academics, as typically disproportionately involved in household and pastoral activities, that will be most disadvantaged by the home-based and online LTA transition, and vulnerable to a ‘maternal wall’ (Minello 2020) unless COVID-19 also manages to reverse a performative work culture:

*Balancing teaching online- 24 hours a week with a 2-year-old at home on my own is a huge challenge. This is disrupting the learning environment not only for my students, but also my son. My professional and personal role are blurring and I have not got time to successfully do both the best of my ability.*

Massive work-intensification of this kind was also represented as a skills *cul-de-sac* and impediment to lecturers’ capacity building, with respondents claiming being time-starved and deprived of the necessary space and (timetabled) affordances that might otherwise allow them to cultivate digital competencies—which some were also clearly willing to embrace. Instead, respondents echoed how online migration was contributing to their deprofessionalisation as pedagogues and also to the collapse of responsible work-allocation by their institutions:

*I would like to engage in more training to become a more tech-savvy lecturer, but with no time to prepare, play, explore the technologies available, at the moment I am merely stressed out of my wits, as are my students. At my own institution, I don't think that much will change for my role, except perhaps even more responsibility and administration.*

Hesitation if not direct objection to online migration was also articulated by respondents in the terms, sometimes of quite profound pedagogical scepticism and a perception of online LTA as not only conflicting with their teaching beliefs but being at odds with desired learning outcomes and needs specific to certain disciplines. Respondents, furthermore made plain their unwillingness to adapt or even entirely redesign material given the weight of existing pressures (and lack of training opportunities) and moreover, we would speculate, limited time before a new academic year and fresh student intake:

*I’m being asked to teach online, a modality that I think leads to crap outcomes in my field because it is based on critical thinking and conversation, not rote memorization, and I have no real training in online course design. I have no interest in teaching like this anyway, and there is no possible way I am going to invest in completely re-doing this course on the fly anyway.*

The timing of online migration was again considered in the terms of research and the urgency of universities response in moving LTA online and relatedly a massively enhanced administrative burden over summer months normally prioritised for research resulting in its cessation:

*The huge increase in workload (both learning and applying new technology and managing student queries/expectations as they do the same) is killing my research. These changes are lasting far beyond the usual term time - for example, we are expected to find and upload many additional online resources to support online assessments, and engage in pastoral care (including assisting students adapt to online learning and assessment) at a time when we would normally be conducting research. The summer term (when we might be able to catch up on research time or consider ways to improve our current provision) is going to be dominated by frantically re-designing modules to be more amenable to online teaching and assessment, and completing all the paperwork required to support these changes (and even then, there isn't enough time to ensure a cohesive and balanced approach across the whole course). Basically, any thoughts of getting research done or presenting a cohesive programme to students next year is disappearing.*

The impact of COVID-19 on research was also recognised in the context of university premises having been closed and many/most institutions prohibiting national and international fieldwork. As one of our respondents succinctly put it, ‘My research is field based and now in the toilet for the year’.

A trend of research cessation alongside intensifying LTA commitments caused by the COVID-19 pandemic was commonly associated by respondents as further amplifying a well-established trend of occupational precarisation in higher education (cf. Courtois and O’Keefe 2015), and directly causing career stasis and an overall contraction if not flat-lining of the academic labour market. For some, emergency online migration represented an inescapable diversion from producing research outputs and consequently a potentially insurmountable barrier to achieving job security:

*I’m up for tenure soon and am under immense pressure to get my monograph out at an excellent publisher in order to keep my job. This was supposed to be my term(s)/summer to do so, as I have so far been prevented in making sufficient progress due to demands of teaching and programme administration. I honestly have no idea how, given the massive deluge of new demands about teaching, admin, and online preparation and contingency planning, I will get that done. The university has given no guidance on how this will all effect the timing of progression, which is completely terrifying.*

Many respondents also spoke of elevated precarity for casualised academic staff—whose redundancy might be more easily rationalised—as a group particularly susceptible to the seemingly imminent threat of job losses, linked to predictions of lower levels of student recruitment. Such observation might also be treated as an indictment of academics’ ill confidence in the quality, and their reticence in transitioning to digital education, which they also fear in the long-term as a cost-saving initiative:

*As a worker on a precarious contract, I will be under increasing threat of job loss in response to economic implications of loss of recruitment. Lower student satisfaction will result in lower NSS*^2^*results and negatively affect recruitment. Senior management have deemed online teaching lightens the workload for academic staff (it does not), and have already encouraged department heads to let associate/part-time staff go*

Unmistakable in respondents’ accounts is an over-riding fear that universities—and potentially governments too—will turn the tragedy of the coronavirus and its inescapable economic impacts as a means to legitimately carry through preexisting plans for cost-cutting (cf. Watermeyer et al. 2020):

*The experience of the crisis may be used as a pre-text to move more teaching online in order to increase student fee income while decreasing overhead costs (e.g. for buildings that are already bursting at the seams due to larger and larger student intakes).*

*I worry that as we seem to have proved we can deliver everything online that this will become the new normal and the government will use it as an opportunity to scale back universities in the UK.*

Instances of non-renewal of fixed-term academic contracts has already manifest in some UK institutions. Such action regrettably has inevitably not only hit hardest the most vulnerable members of the academic workforce but placed further stress on an already hyper-competitive and analogously job-impoverished labour market, which would appear to be now entering a period of prolonged stasis with a moratorium advertised in many universities on recruitment.

An assumption, however, among many respondents that students will find an online LTA model less attractive than a traditional method of delivery and thus ‘speak with their feet’, ignores historical evidence of increased HE uptake at times of economic recession. This take on student recruitment was nevertheless supported by albeit far fewer respondents who contemplated ‘*that home recruitment will hold up as fewer will want to enter the workplace in what is likely to be a huge recession*’*.* Somewhat conversely the suggestion was also made that many students would seek to postpone their studies until such time and on the assumption that traditional in-person tuition would return—perhaps rather optimistically—within a year:

*I can see a lot of students opting to defer by a year to arrive in 2021 when they will be able to have a face-to-face university experience from the beginning.*

Some even suggested that the speed by which certain countries were able to return to a traditional model of LTA would have a bearing on student recruitment, particularly of international students:

*In terms of international recruitment, this is even more influenced by differential developments in the virus in different countries - for example, if the UK were back to face-to-face teaching in September, and the US was not, we could see a boom in overseas students coming to the UK.*

In overview predictions of workforce streamlining (rationalised by technologically enabled economising), a swelling in the numbers of unemployed academics, deteriorating work conditions (especially for those whose work-intensification might only further augment with depletion of staff numbers) and heightened occupational precarity and insecurity, respondents also addressed the risk of brain-drain and potentially even, a lost generation of academic talent, unable to find—or disincentivised to find—a first perch on the academic job ladder:

*When the 2008 recession hit, they delayed granting tenure for a few years; I hope I don't lose my awesome younger colleagues over this.*

*The job market is going to collapse. I'm so sorry for PhD students and early career researchers.*

### Affordances

While the vast majority of accounts from our academic respondents revealed an altogether downbeat diagnosis of the impacts of online migration, there were some among our sample who were more optimistic. Their optimism, significantly, was the inverse of the fatalism articulated by many others, in that they perceived in the tragedy of COVID-19 and enforced online migration not the decline of the higher education sector but affordances and an opportunity, long overdue if perhaps previously difficult to exploit—*HE has been long overdue a complete overhaul*—*for* accelerated modernisation—as one respondent commented, ‘*We will probably achieve more in 3 months than we would have in 3 years. In Lewin’s change management model we have ‘unfrozen’ at an incredible rate*’*.*

Moreover, online migration might be perceived as a step-change in the professionalisation of academics as teachers far exceeding the tokenism of pedagogic credentialism and other recent efforts to incentivise best or better practice:

*The sector may re-emerge better off for having had some of its underlying assumptions about learning and teaching challenged.*

Furthermore, for these technology advocates, online migration represented far more than just a pedagogical sticking plaster and instead an unparalleled opportunity for pedagogical reinvention. It also appears to provide belated confirmation for the importance of technology experts in guiding the evolution of universities’ digital education provision:

*The COVID-19 outbreak has highlighted the skills and expertise of TEL teams and Educational Technologists globally - such a shame that it has taken such an awful situation to flag their worth.*

While only a minority—whose enthusiasm might also reflect sample bias, given *n* = 119 respondents stated computer science as disciplinary affiliation—these accounts are important for reflecting a view of the necessity for change and its cognate advantages, primarily of efficiency and enhanced control, and resistance to ‘going back’:

*The transition to full remote teaching is making my job VASTLY EASIER. I can prepare all my material until the last moment in the quiet of my home. I can precisely control the teaching environment, and record and dissect/debrief/improve. For me this is a RENAISSANCE. Should we go back to physical teaching, I will ask my leadership whether I can continue to do a fully online section.*

While many of our respondents had previously spoken of online migration causing social disconnection—especially for those without adequate access to online technologies—among those advocating its benefits, a turn towards digital education was greeted conversely as an opportunity for enhanced social connectivity and inclusivity: ‘*my feeling is that the whole ethos of HE is likely to become more ‘modern’ and ‘innovative’ and as a result of these much more inclusive and accessible*’*.*

The benefits of connectivity were also seen to apply to faculty members and online migration forcing the hands of those—typically more established academics—in not only adopting new technologically facilitated methods of LTA but placing their trust in younger, less experienced yet ostensibly more technologically adept colleagues. In such terms, COVID-19 and online migration was seen for bringing academic communities closer together, ironically at a time when they are physically most apart:

*This disruption has had at least two strong internal advantages: - Everybody has finally made an effort to transition to online learning. Older faculty have had to rely on the expertise of younger faculty (whom they were quick to dismiss until now). Overall my department and community has been talking more to each other. I wish COVID-19 did not come with horrible impact to many people, otherwise I would be tempted to see it as a blessing of sorts.*

## Discussion

The results of our survey show academics bruised by their experience of emergency online transition and distrustful of a more prolonged and substantial embrace of digital pedagogies by their institutions; confirming the findings from previous studies that signpost academics’ hesitancy towards and suspicion of higher education’s digitalisation (Marshall 2018; Selwyn 2017; Williamson 2020). Their accounts are a story of trauma in the face of pandemic and of profound professional and personal disruption. They also constitute a critical history of the lives of academics in the milieu of higher education’s global—and seemingly irreversible—transformation. We find a history of professional dysfunction and disturbance, of inequality, exploitation and neglect; of confidence and trust abused and squandered; of disempowerment, displacement and marginalisation; of self-concept on trial and in tatters; of vulnerability and helplessness; and of the loss of a much maligned past superseded by the perceived machinations of digital dystopia and threat of professional oblivion. This is a history peppered in angst, confusion, fear, resentment and a peculiar dichotomy of both dreading the unknown yet resenting the relative assurance of what will come to pass. Yet these are well known aspects of academics’ recent history and while COVID-19 is undeniably a story of abrupt and violent change, it is hardly *sui generis*. It instead represents the total assimilation of many threads of academic discontent in the face of professional revolution, visited upon in this survey, which together positions many members of the UK higher education community at a point of no return facing a future they no more desire yet must face. The COVID-19 crisis appears thus to have dually quickened the inevitability of technological change or authority of technological determinism and supercharged a sense of existential panic among academics—many of whom appear now snared in the headlights of digital disruption. Yet, the weight of a recent history of disconsolation forces us to ask quite how much it has caused to prejudice and distort many of our respondents’ views of digital pedagogy and the extent to which their state of vulnerability causes any further change to their role to be viewed only through the lens of precarisation.

In this sense, we are obligated to question the extent to which digital pedagogies are in the COVID context and a rushed online migration, afforded a fair trial. We might reflect that while some of the technology advocates among our sample identify an opportunity in the current pandemic to force the case for digital transitioning, the aggressiveness of the case being made in an emergency context is bound to negatively prejudice the views of many already wary and more so, over-burdened and disconsolate workers. Moreover, while the current context of online migration arguably provides for only the very slimmest and superficial of encounters for academics with digital pedagogy, its introduction at a time of extreme stress and uncertainty is fixing a highly reductive and recusant view in many cases limited to a notion of ‘posting things online’ and of technology causing role-invalidation. In the latter case, academics’ fears of the impact of a digital revolution on their jobs are already being confirmed with some institutions allegedly now permanently moving substantial chunks—up to a quarter—of their teaching portfolio online.^3^

There are other good reasons for academics’ misgivings. These we would relate to prospective prolonged work-intensification in the face not just where job cuts are made, but suspicion that the necessary investment in technologically enhanced or facilitated learning in universities might not necessarily be made. There must a reasonable chance and equally profound danger that the financial challenge presented by COVID-19 to the sector—and the potential return of a politics of austerity in the distribution of public funds that resonates with the 2008 global crash—will mean that a digital ‘renaissance’ will fail to materialise, unless only among the most well-off institutions or as represented by those already digitally converted. And without a sustained and substantive commitment to resourcing digital transition the kinds of dumbed-down pedagogy associated with the current emergency online migration will likely remain the status quo and problem of academics—as articulated by our respondents. In such event, reductive views on the contribution of digital pedagogies will surely only ossify and with them concerns as to the quality and attractiveness of university tuition, its value for money (and the willingness of students to pay high fees), and its workforce contribution via graduate talent (see also Lederman 2020). There are clear global market implications for online migration too, with student recruitment likely becoming even more competitive and a counter-logic that those countries more able to quickly recover from COVID-19-related closures and return to a traditional model of university tuition being most likely to benefit. We might also reasonably speculate that only those institutions most able to provide a quality online experience—or with the most secure and recognisable branding—will sustain market advantage, and thus approaches to marketing excellence in the recruitment of students will necessarily have to change (see also Lloyd Clark 2020). Concurrently, if job losses as a consequence of institutional cost-cutting follow through in large numbers there will likely be an overall contraction of the higher education sector—though this will be unequally concentrated and vary cross disciplinary, institutional and country contexts—and therefore even greater demands made of those remaining, already struggling to contend with the intensification (Kenny 2017), unbundling (Macfarlane 2010) and reassembling (Lewis and Shore 2018) of their roles. Precarisation, prolertarianisation and performativity will continue to proliferate. Competitive accountability will not crack but concretise as the guiding principle of academic labour. Analogously, access to and progression within an academic career will be become even more tenuous; a trend of casualisation may further widen, and the Academy may be a whole generation of scholars worse off.

At the same time, our respondents’ accounts intimate that a pre-existing crisis of mental health in universities may worsen and not just for students but academics too—struggling to manage increased pastoral demands with the needs of home, and forfeiting their right to work-life balance. Academics connection with colleagues may also deteriorate—though some of our respondents spoke of technology and the pandemic more broadly in actually helping to mend gaps in fractured departments and boosting collegiality; enabling perhaps a ‘kinder research culture’ (Derrick 2020). Students at the same time are predicted to suffer—if not already—from the potential effects of pedagogical dislocation and becoming disengaged from their studies and learning communities. The student experience, especially for undergraduate cohorts will also irrevocably change if the current situation is to persist—and so too potentially a pressure of their continuing stay with parents—though they will likely be better off financially. The damage to local and national economies as a consequence of them not having to physically attend campuses will be huge (London Economics 2020).

There are a depressing abundance of afflictions identified by our respondents associated with emergency online migration that overshadow the potential affordances of digital pedagogies. Moreover, there are clear signs that the affordances on offer may not materialise given the severity of potential cuts to the sector. However, there may be some consolation and hope that current predictions related to a massive fall off in student recruitment, particularly at an international level, may be compensated for by the effects of global recession (HEPI 2020), and a corresponding influx of new students—though the majority of these will likely be home-based and lower fee-paying.

The impact of COVID-19 and the emergency online migration of university communities is undeniably huge. Nevertheless, we are only at the earliest of beginnings of recognising and understanding these impacts on the role of academics and the future of global higher education. There are many follow-up questions to pose. What is the extent of the challenge of private higher education providers to public universities in the context of digital pedagogies and online migration? How are academics responding to the broadening of their pastoral role and how in turn are universities supporting them? What now, of an opportunity of a humane response to the welfare of the higher education community and what if any are the consequences of COVID-19 to the dominance of neoliberal values? How many universities will go (fully) digital? Quite how great will the impact of COVID-19 be on non-COVID-related research? Where our survey has demonstrated variation of academics by disciplinary field in their response to emergency remote learning, which disciplines will be most vulnerable, disadvantaged and in need of support in any long-term pivoting to digital pedagogies? And of course, how long will it be before we know the full impacts of these changes? These are but a few huge questions that require urgent address in a time, like no other, where the future of higher education—and academics especially—appears in jeopardy. Moreover, these are questions to be followed through in research that longitudinally interrogates the change-basis of COVID-19 across international higher education systems, and which provides, beyond this initial snapshot, a fuller and deeper global analysis of comparative trends pertaining to digital transitions. There is thus urgency both in terms of formulating an exit-strategy from the COVID-19 crisis—though for this we anticipate no quick-fix—and yet also consensus of exactly what the higher education sector will be exiting to.

## Footnotes

1 The Open University is the UK’s major distance learning higher education provider: http://www.open.ac.uk/about/main/

2 NSS is the UK’s *National Student Survey*, an annual survey completed by final year undergraduate students in the UK’s home nations of England, Scotland, Wales and Northern Ireland, which captures perspectives on the quality of degree programmes.

3 https://www.theguardian.com/education/2020/apr/17/lecturers-condemn-durham-universitys-plan-to-shift-degrees-online
